# Human Physiology

**Published:** 1851-01

**Authors:** 


### ARTICLE V.

*Human Physiology.* By ROBLEY DUNGLISON, M. D., Prof. of the Institutes of Medicine in Jefferson Medical College, Philadelphia; Vice President of the Sydenham Society of London; Secretary to the American Philosophical Society, etc., etc. With nearly five hundred illustrations—seventh edition, thoroughly revised, and extensively modified and enlarged. In two volumes. Philadelphia, Lea & Blanchard, 1850, pp., 1416, 8 vo. (From the publishers, for sale by S. C. Griggs & Co., Chicago.)

As this is a standard work already well known to the profession, we have merely to say that the author has bestowed much labor on this edition, in order to bring it up to the present state of Physiological knowledge, and has enriched it by the addition of numerous illustrations. We cannot but think that the motto prefixed to the work is unfortunate.

"Vastissimi studii primas quasi lineas circumscripsi." It is enough to discourage the most resolute beginner in physiology to be informed that these portly volumes are merely *primas quasi lineas.* M.

